# Clavipectoral Fascial Plane Block for a Permanent Pacemaker Insertion in a Claustrophobic Patient With a History of Substance Abuse: A Case Report

**DOI:** 10.7759/cureus.88726

**Published:** 2025-07-25

**Authors:** Gade Sandeep, Subrata K Singha, Sharek Nazir, T C Arun, Anil Gupta

**Affiliations:** 1 Cardiac Anesthesia, All India Institute of Medical Sciences, Raipur, Raipur, IND; 2 Anesthesiology, All India Institute of Medical Sciences, Raipur, Raipur, IND; 3 Anesthesiology and Critical Care, All India Institute of Medical Sciences, Raipur, Raipur, IND

**Keywords:** general anesthesia, opioids, pacemaker, regional anesthesia, substance abuse

## Abstract

Permanent pacemaker implantation in patients with severe claustrophobia and a history of substance abuse presents unique anesthetic challenges. The balance between ensuring patient comfort and avoiding the risks associated with systemic sedation or general anesthesia requires careful planning. In this report, a regional anesthetic technique, the clavipectoral fascial plane block, was used to facilitate pacemaker insertion in a high-risk individual without the need for sedatives or opioids. The anesthetic approach was supported by preoperative psychological counselling and tailored modifications to the sterile draping, allowing the patient to remain oriented and cooperative. This case underscores the value of combining regional anesthesia with empathetic, patient-centered strategies in complex perioperative scenarios.

## Introduction

Permanent pacemaker implantation (PPI) is routinely performed under local anesthesia, and often supplemented with minimal sedation or monitored anesthesia care (MAC) to ensure patient comfort and immobility [1]. This conventional approach involves local anesthetic infiltration at the incision site, along with titrated doses of sedatives such as midazolam, fentanyl, or propofol [2].

However, in patients with complex psychological or substance use histories, such as severe claustrophobia or chronic drug abuse, local infiltration and sedation may be inadequate or unsafe. Sedatives and opioids in this group can lead to unpredictable pharmacodynamic responses, increased risk of respiratory depression, paradoxical agitation, or drug interactions [3]. Claustrophobia during awake procedures also complicates intraoperative management, especially when extensive sterile draping induces anxiety or panic [4].

The clavipectoral fascial plane block (CPFB) targets nerves traversing the clavipectoral fascia, including the lateral and medial pectoral nerves and intercostal branches (T2-T6), providing effective sensory blockade of the infraclavicular region. Although its use is well-documented in clavicle surgery, reports on its application for PPI remain limited [5]. This case adds to the emerging evidence supporting CPFB as a safe, effective, and technically feasible alternative for anterior chest wall anesthesia.

We present the case of a 56-year-old male patient with idiopathic complete heart block, claustrophobia, and a history of polysubstance abuse who was scheduled for PPI. In this patient, CPFB was used as the sole anesthetic technique, combined with psychological counselling and draping modification, to avoid systemic sedation or general anesthesia. 

Informed consent for publication was obtained from the patient as well as from a first-degree guardian in accordance with our institutional protocol, considering the patient’s history of substance use.

## Case presentation

A 56-year-old male patient, weighing 50 kilograms, presented with a three-month history of frequent dizziness. A 12-lead electrocardiogram revealed a complete heart block with a ventricular rate of 34 beats per minute. Transthoracic echocardiography showed no structural cardiac abnormalities. As an initial emergency intervention, a transvenous temporary pacemaker was inserted via the right femoral vein. With a provisional diagnosis of idiopathic complete heart block, the patient was scheduled for a PPI.

The initial attempt at PPI was planned under local anesthetic infiltration of the left infraclavicular region, without sedation. However, the patient became acutely anxious and uncooperative during surgical draping due to severe claustrophobia, leading to abandonment of the procedure.

During the pre-anesthetic evaluation for the second attempt, the patient disclosed a history of substance abuse, including chronic use of cocaine, tobacco, and alcohol, with the last use reported four days prior. The patient had a history of chronic substance use, including tobacco use of approximately 10 pack-years, daily alcohol consumption of approximately 60 grams of ethanol per day, and recreational cocaine use once or twice per week in small amounts. At the time of presentation, the patient exhibited no signs or symptoms of withdrawal.

Due to concerns about unpredictable sedative responses, myocardial irritability from cocaine use, impaired drug metabolism from alcohol, and increased respiratory risk from chronic smoking, we planned a regional anesthesia approach using a CPFB, thereby avoiding systemic sedation. The patient's biochemical and hematological investigations were within the normal limits.

In case the block was found to be inadequate intraoperatively, the anesthetic team had planned to convert to MAC using low-dose ketamine (0.2 mg/kg bolus followed by 0.2 mg/kg/hr infusion) while maintaining spontaneous ventilation.

**Figure 1:**
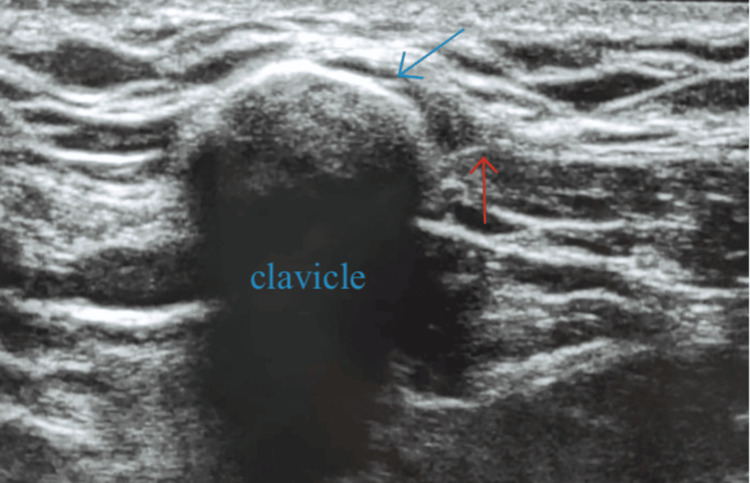
Image showing the clavipectoral fascia and the clavicle (the blue arrow shows the separation of fascia and the red arrow shows the needle)

On the day of the procedure, the patient received detailed counselling regarding the anesthetic technique and the surgical process. After establishing intravenous access with a 20G cannula and attaching monitors as per American Society of Anesthesiologists standards, which included a five-lead ECG and non-invasive blood pressure and pulse oximetry, the procedure was performed under strict aseptic precautions. Under ultrasound guidance (SonoSite Edge II Fujifilm, Bothell, WA) using a high-frequency linear probe (2-5 MHz), a CPFB was administered with 20 mL of 0.25% bupivacaine combined with 8 mg of dexamethasone (Figure 1).

Sensory blockade was assessed using the pinprick method and confirmed 15 minutes after block administration, indicating adequate onset before surgical incision. The CFPB provided effective sensory coverage from T2 to T6 dermatomes, with a Numerical Rating Scale (NRS) pain score [6] of zero reported throughout the procedure. The patient was followed up for 24 hours postoperatively, during which the NRS score remained below four with regular intravenous paracetamol for analgesia. The cardiologist did not have to supplement with any local infiltration for analgesia.

**Figure 2:**
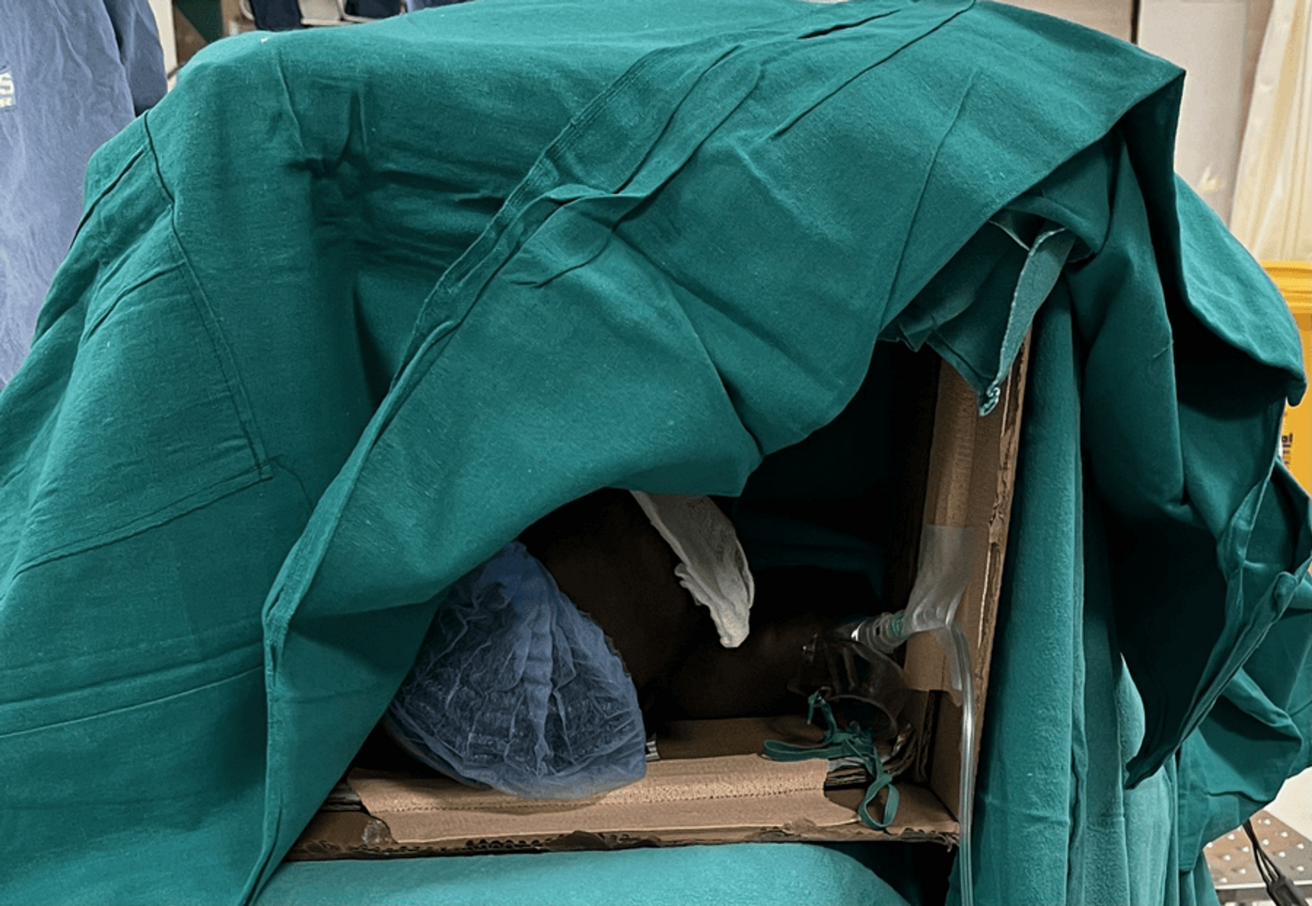
Image showing the modification of drape to allay the patient's anxiety (his eyes were covered for the image)

To minimize claustrophobia-related distress, sterile drapes were modified to keep the patient’s face and immediate environment visible, helping him remain oriented and comfortable throughout the procedure (Figure 2).

The entire procedure was completed within one hour without complaints of pain, anxiety, or discomfort. The patient remained cooperative and hemodynamically stable throughout. Post procedure, the patient was monitored in the recovery area and received intravenous paracetamol, 1 g every eight hours, for analgesia. There were no immediate complications, and the patient expressed satisfaction with the anesthetic experience.

## Discussion

PPI is typically performed by cardiologists using local infiltration of lignocaine at the incision site, often without sedation. However, in certain populations, particularly those who are anxious, uncooperative, or have a history of substance abuse, this approach may be insufficient or potentially unsafe. In such cases, anesthesiologists can play a pivotal role by providing regional anesthesia, using techniques that ensure adequate surgical anesthesia while minimizing the risks associated with systemic sedatives and opioids [1].

The clavipectoral fascia is a dense connective tissue layer located between the clavicle and the pectoralis minor muscle, lying deep to the clavicular head of the pectoralis major [7]. The CPFB is a relatively new regional anesthetic technique that has shown promise in surgeries involving the anterior chest wall, including PPI. This block targets the sensory innervation of the infraclavicular region, primarily through the medial and lateral pectoral nerves, the intercostobrachial nerve, and the upper intercostal nerves (T2-T6) [5].

Although the present case proceeded without complications, potential risks such as vascular puncture, pneumothorax, and local anesthetic systemic toxicity (LAST) must be acknowledged, given the anatomical proximity of the block to the subclavian vessels and pleura. These risks, however, can be significantly minimized when the procedure is performed under real-time ultrasound guidance using a precise, in-plane approach by experienced practitioners.

CPFB involves depositing local anesthetic between the clavipectoral fascia and the clavicle, a more superficial and cranial location compared to other fascial plane blocks like pectoral nerve block (PECS) and serratus anterior plane (SAP). This anatomical advantage results in a markedly lower risk of pneumothorax, making it a safer alternative in select patients [8]. In comparison, PECS I and II blocks are designed to anesthetize the lateral and medial pectoral nerves and intercostal nerves, but are generally performed at a deeper level and may not consistently cover the medial infraclavicular area relevant to pacemaker pocket creation. The SAP block, although effective for lateral thoracic wall analgesia, does not reliably target the nerves innervating the anterior chest wall [9]. CPFB, by directly anesthetizing the terminal branches of the pectoral nerves within the clavipectoral fascia, may therefore offer a more focused and effective regional block for procedures limited to the clavicular and infraclavicular region, such as pacemaker implantation.

Unlike local infiltration by cardiologists, CPFB offers more complete dermatomal coverage of the surgical field and avoids the need for repeated injections. Additionally, in uncooperative or anxious patients, there is a risk of exceeding the maximum safe dose of local anesthetic during blind infiltration, increasing the potential for LAST. Anesthesiologists, trained in ultrasound-guided regional techniques and the recognition of LAST, are better equipped to manage such risks.

This case was further complicated by the patient’s history of polysubstance abuse, including cocaine, alcohol, and tobacco. Cocaine sensitizes the myocardium to catecholamines and increases the risk of perioperative arrhythmias and hemodynamic instability. Chronic alcohol use may impair hepatic metabolism and exacerbate the effects of sedatives, while chronic smoking can blunt the response to intravenous anesthetics and increase the risk of respiratory complications. Taken together, these factors made both conscious sedation and general anesthesia high-risk options. Even minimal sedation could have triggered agitation, respiratory depression, or adverse drug interactions [3].

Claustrophobia posed an additional challenge. The sterile field for PPI typically requires extensive draping, which can exacerbate anxiety in claustrophobic patients. While general anesthesia is sometimes used to bypass such challenges, it was better avoided in this case. Instead, a non-pharmacological anxiolysis approach was adopted. Psychological counselling, procedural rehearsal, and a modified draping technique allowing visual orientation were successfully employed to manage the patient’s distress [10].

Importantly, a contingency plan was in place: if the CPFB failed, the team planned to initiate MAC with a low-dose ketamine infusion while maintaining spontaneous ventilation. The decision to consider low-dose ketamine as a backup sedation plan was based on emerging evidence supporting its potential benefit in patients with cocaine use disorder, including reduction in craving and harm [11]. Additionally, ketamine’s rapid onset of action offered a practical advantage over agents like dexmedetomidine in the event that procedural sedation became urgently necessary. This ensured patient safety while avoiding escalation to general anesthesia.

## Conclusions

This case highlights how the integration of regional anesthesia with patient-specific psychological and environmental strategies can enable safe and comfortable performance of procedures that typically require sedation or general anesthesia. While CPFB provided reliable surgical anesthesia for PPI, non-pharmacological measures such as preoperative counselling and modified draping played an equally vital role in managing the patient’s claustrophobia and anxiety.

However, the use of CPFB remains infrequent, and larger clinical studies are warranted to compare its efficacy with more commonly used techniques such as PECS I, PECS II, and SAP blocks.
